# Artificial Intelligence Methods for Smart Cities

**DOI:** 10.3390/s24082615

**Published:** 2024-04-19

**Authors:** Alessandro Sebastian Podda, Salvatore Carta, Silvio Barra

**Affiliations:** 1 Department of Mathematics and Computer Science, University of Cagliari, Via Ospedale 72, 09045 Cagliari, Italy; 2 Department of Electrical and Information Technologies Engineering, University of Naples “Federico II”, Corso Umberto I 40, 80138 Naples, Italy

## 1. Introduction

In recent years, the concept of smart cities has garnered increasing attention as urban areas grapple with the challenges of population growth, resource management, and infrastructure optimization. Smart cities leverage innovative technologies to enhance the quality of life of their residents, promote sustainability, and improve the efficiency of urban systems [1,2]. Central to the realization of smart city initiatives are advancements in Artificial Intelligence (AI) [3,4] and the Internet of Things (IoT) [5,6,7], which offer unprecedented opportunities for data-driven decision making, automation, and real-time monitoring.

This Special Issue aimed to collect the latest developments in AI and IoT technologies within the context of smart cities. The published articles, in addition to having received positive feedback from the scientific community so far, provide valuable insights into the diverse applications of AI, the IoT and networks in addressing key urban challenges and optimizing city operations. In particular, a common aspect among the different studies is the emphasis on anomaly detection frameworks involving machine learning, deep learning, and computer vision methods, still widely adopted in the literature in a variety of application domains, from healthcare [8,9,10] to cybersecurity [11,12,13] to fintech [14,15], as revolutionizing solutions for urban surveillance [16,17], traffic management [18,19,20], and public safety [21,22].

Moreover, among the received contributions, the synergies between AI and IoT technologies and their combined potential to drive transformative change in urban environments emerge, without disregarding the challenges and ethical considerations associated with the widespread adoption of these technologies, including data privacy, security vulnerabilities, and algorithmic biases.

## 2. Overview of Published Articles

The first contribution published in the Special Issue is a research article by Bassetti et al. (contribution no. 1) focused on the acquisition of motion data within vehicular environments through smartphone technology. They show how smartphone sensors are capable of gathering data across diverse contexts, at minimal or negligible expense given the widespread ownership of mobile phones. However, the varying placement of smartphones inside cars presents challenges in interpreting the captured data, owing to the ambiguity surrounding their orientation. To this purpose, they propose a methodology aimed at automatically realigning smartphone-generated data captured within vehicular settings to conform to a standardized orientation, which integrates a blend of least-square plane approximation techniques, machine learning algorithms, and rotation matrices. The solution was tested in a simulated environment, and the experimentation showed that their method performs well and shows promise for future applications in real-world scenarios.

Sticking with the context of the vehicular environment, Sadli et al. (contribution no. 2) propose a low-cost solution for lane-level localization, i.e., a real-time measurement method of a vehicle’s position relative to the median lane. Such a solution employs a vision-based system to improve the performance of simple GPS-based detection, and aims to provide a viable alternative to LIDAR systems, which are more reliable and accurate but have a significantly higher costs of access. The results obtained showed a significant reduction (by about −50% globally) in deviations compared to the GPS-only benchmark.

Noh et al.’s investigation (contribution no. 3) shifts the focus to pedestrians and crosswalks by enhancing comprehension of road users’ hazardous actions and aiding decision makers in fostering safer road infrastructures through informed decisions. It presents a novel approach aimed at discerning potential risky behaviors in smart cities, through the analysis of CCTV footage deployed along roadways. Their goal is to automatically extract behavioral features of vehicles and pedestrians that influence the probability of collisions, particularly within crosswalks, through a combination of scene partitioning techniques, object detection and tracking methods based on deep neural networks, and heuristics for the task of feature extraction. The study includes an extensive validation phase, which has made it possible to extrapolate a broad spectrum of events and circumstances that the authors believe to be of particular use in determining events that may cause potential accidents for the purpose of future analysis and research, as well as in land use and strategic planning.

While the previous contributions focused on issues related to traffic monitoring, vehicle data, and more general aspects of viability in smart cities, Ingle and Kim (contribution no. 4) enter the sharper perimeter of video surveillance, addressing the problem of identifying abnormal objects, e.g., weapons, in the monitored scene, a subject that is reflected in the growing security needs of our time. Indeed, the widespread adoption of video surveillance systems for object detection has led to the need to monitor multiple cameras to detect abnormal behavior, which is particularly challenging in setups with numerous cameras, many of which operate under resource constraints. To address these challenges, the authors presented a method based on convolutional neural networks, tailored for the real-time detection of firearms and knives. They also reported detection tests showing promising results, achieving an average precision of 97.50% on various datasets and 90.7% on multi-view cameras, even in resource-constrained settings.

The study by Chen et al. (contribution no. 5) moves towards the detection of fatigued driving. As acknowledged by the authors themselves, this is a research topic that has been widely addressed in the literature; however, in their work, they place emphasis on an entirely novel and relevant element: the cumulative effect of time on human fatigue. To this purpose, they introduce a new model combining back propagation neural networks and time cumulative effects to tackle fatigue detection issues, validated with experimental data. Specifically, they performed face detection, face tracking, and facial landmark detection, and then exploited a neural network trained on selected features, including, e.g., eye closure duration and yawn frequency, to identify fatigued driving. As previously mentioned, the groundbreaking element of this work lies particularly in the use of a model incorporating time accumulation, by employing a sigmoid function, to segment drivers’ fatigued states from recorded videos. Compared to traditional models, the aforementioned approach leads to promising results, with an average improvement of more than 5% considering both cases with and without facial expressions.

A work that sharply highlights what smart cities can offer through the integration of artificial intelligence techniques within existing video surveillance systems is that of Nadeem et al. (contribution no. 6). Their investigation introduces a sophisticated mechanism aimed at tracking missing individuals within crowded gatherings. The innovative methodology presented is not only capable of locating individuals within low-resolution images of large assemblies, but relies on a novel geofence set estimation algorithm that streamlines the search effort by efficiently narrowing down the search area. The outcomes of their extensive testing reveal a high degree of reliability and satisfactory performance, and this solution could potentially emerge as a valuable asset for civil society.

Differently from the previous works, contribution no. 7 from Ismail and Buyya is a review paper that extensively espouses a comprehensive perspective of 6G networks, encompassing applications, technologies, and infrastructures oriented toward the development of intelligent, cohesive, and decision-centric digital smart city ecosystems. The motivation for their study arises from the exacting demands for quality of service, availability, and dependability essential for meeting service-level agreements (SLAs) for end-users. The contribution, which emphasizes the integration of AI within 6G network protocols and operational frameworks, is threefold: first, the authors delineate the evolutionary trajectory of wireless network generations, encapsulating the progression from 1G to AI-enabled 6G, elucidating the concomitant evolution of their applications; second, they provide a taxonomy of technology-driven smart city applications; and third, they show prospective directions for research in this area.

Returning to the realm of research articles, the two subsequent works published in the Special Issue are both oriented towards indoor surveillance. The first one, by Lan and Yoon (contribution no. 8), introduces a method for detecting anomalies in human movement within indoor spaces by analyzing trajectories and by concentrating on urgent situations like security threats and accidents. Their method involves two steps, the first one being to group the data into clusters and the second one being to evaluate the abnormality of retrieved trajectories by also employing a novel trajectory similarity metric that they name the Longest Common Sub-Sequence using Indoor Walking Distance and Semantic Labels. This method has been found to be remarkably effective in experimental tests on two major literature datasets, the *MIT Badge* and the *sCREEN*. The second study, by Abbasi et al. (contribution no. 9), addresses the more general task of detecting and tracking multiple individuals in indoor scenarios. Their method consists of a low-level, multi-input ensemble which mainly employs a Neuromorphic Vision Sensor to collect the data and an extensive experimental phase that involves selecting the appropriate features to feed different deep learning networks deployed to track the individuals. Their findings revealed significant disparities between the input features of optimized neural network backbones: event-based frames emerge as the preferred input feature type, although they acknowledge the necessity for further validation through additional studies.

The last study published in the Special Issue is by Hayee et al. (contribution no. 10). It introduces a novel method which relies on Convolutional Neural Networks, integrated with a customized classifier based on Fisher’s discriminative least squares regression, to address the challenge of recognizing the make and model of vehicles. Not only has their method been evaluated on publicly available datasets, demonstrating competitive performance compared to existing techniques, but it also enhances the classification accuracy of such a task and proves to be suitable for real-time applications.

In conclusion, this Special Issue highlights the critical role of AI and the IoT in outlining the future of smart cities; it also offers valuable insights for researchers, policymakers, and urban planners seeking to harness the full potential of these transformative technologies. Through interdisciplinary collaboration and continuous innovation, we envision a future where AI- and IoT-enabled smart cities foster sustainable development, resilience, and prosperity for all residents.
